# Differential histopathologic parameters in colorectal cancer liver metastases resected after triplets plus bevacizumab or cetuximab: a pooled analysis of five prospective trials

**DOI:** 10.1038/s41416-018-0015-z

**Published:** 2018-03-13

**Authors:** Chiara Cremolini, Massimo Milione, Federica Marmorino, Federica Morano, Gemma Zucchelli, Alessia Mennitto, Michele Prisciandaro, Sara Lonardi, Alessio Pellegrinelli, Daniele Rossini, Francesca Bergamo, Giuseppe Aprile, Lucio Urbani, Luca Morelli, Marta Schirripa, Giovanni Gerardo Cardellino, Matteo Fassan, Gabriella Fontanini, Filippo de Braud, Vincenzo Mazzaferro, Alfredo Falcone, Filippo Pietrantonio

**Affiliations:** 1 Unit of Medical Oncology 2, Azienda Ospedaliero-Universitaria Pisana, Department of Translational Research and New Technologies in Medicine and Surgery, University of Pisa, Pisa, 56126 Italy; 2 Department of Pathology and Laboratory Medicine, Fondazione IRCCS Istituto Nazionale dei Tumori - Via Venezian, 20100 Milano, Italy; 3 Medical Oncology Department, Fondazione IRCCS Istituto Nazionale dei Tumori - Via Venezian, 1, 20100 Milano, Italy; 4 Unit of Medical Oncology 1, Department of Clinical and Experimental Oncology, Istituto Oncologico Veneto, Istituto di Ricovero e Cura a Carattere Scientifico (IRCCS), Padua, 35128 Padova, Italy; 5 Department of Oncology, University & General Hospital, Udine - Pz.le S. Maria della Misericordia 15, 33100 Udine, Italy; 6 General Hospital, ULSS8 Berica - East District, 36100 Vicenza, Italy; 7 General Surgery Unit, Azienda Ospedaliero-Universitaria Pisana, Ospedale Nuovo Santa Chiara, Cisanello, 56124 Pisa, Italy; 8 1st General Surgery Unit, Azienda Ospedaliero-Universitaria Pisana, Department of Translational Research and New Technologies in Medicine and Surgery, University of Pisa, 56124 Pisa, Italy; 9 Surgical Pathology Unit, Department of Medicine University of Padua, Padua, via Giustiniani 2, 56126 Padova, Italy; 10 Department of Surgical, Medical, Molecular Pathology and Critical Area, University of Pisa - Via Roma, 67 56126 Pisa, Italy; 11 Department of Oncology and Hemato-Oncology, University of Milan, Milan, Italy; 12 General Surgery and Liver Surgery, Transplantation and Gastroenterology, University of Milan, IRCCS Istituto Nazionale Tumori Fondazione, 20100 Milan, Italy

**Keywords:** Targeted therapies, Colon cancer

## Abstract

**Background:**

Many factors, including histopathologic parameters, seem to influence the prognosis of patients undergoing resection of colorectal cancer liver metastases (CRCLM), although their relative weight is unclear. Histopathologic growth patterns (HGPs) of CRCLM may affect sensitivity to antiangiogenics. We aimed at evaluating differences in histopathologic parameters of response according to the use of bevacizumab or cetuximab as first-line targeted agents, and at exploring the prognostic and predictive role of HGPs.

**Methods:**

We performed a comprehensive histopathologic characterisation of CRCLM from 159 patients who underwent secondary resection, after receiving triplets FOLFOXIRI (folinic acid, 5-fluorouracil, oxaliplatin, and irinotecan) or COI (capecitabine, oxaliplatin, and irinotecan) plus bevacizumab (*N* = 103) vs cetuximab (*N* = 56) in five first-line no-profit clinical trials.

**Results:**

Both major histopathologic response (tumour regression grade TRG1–2, 32 vs 14%, *p* = 0.013) and infarct-like necrosis (80 vs 64%, *p* = 0.035) were significantly higher in the bevacizumab than in the cetuximab group. Achieving major response positively affected relapse-free survival (RFS) (*p* = 0.012) and overall survival (OS) (*p* = 0.045), also in multivariable models (RFS, *p* = 0.008; OS, *p* = 0.033).

In the desmoplastic HGP (*N* = 28), a higher percentage of major response was reported (57 vs 17% in pushing and 22% in replacement HGP, *p* < 0.001) and an unsignificant advantage from cetuximab vs bevacizumab was evident in RFS (*p* = 0.116). In the pushing HGP (*N* = 66), a significant benefit from bevacizumab vs cetuximab (*p* = 0.017) was observed. No difference was described in the replacement HGP (*N* = 65, *p* = 0.615).

**Conclusions:**

The histopathologic response is the only independent determinant of survival in patients resected after triplets plus a biologic. When associated with triplet chemotherapy, bevacizumab induces a higher histopathologic response rate than cetuximab. The assessment of HGPs should be further explored as a predictor of benefit from available targeted agents.

## Introduction

The management of metastatic colorectal cancer (mCRC) patients with liver-limited disease (LLD) is an intriguing challenge for oncologists, as the optimal integration of systemic and locoregional treatments may maximise survival outcomes and even cure a selected subgroup of patients. In the last years, the availability of active systemic treatments and the development of innovative surgical techniques have increased the percentage of potentially resectable patients, thus widening the horizons of pursuable surgical strategies.

Recent guidelines recommend the choice of highly active regimens, able to induce early and deeper tumour shrinkage, as the preferable options for patients with initially unresectable or borderline resectable colorectal cancer liver metastases (CRCLM).^1–3^ Therefore, doublets plus an anti-EGFR monoclonal antibody (only in *RAS* wild-type patients) or the triplet FOLFOXIRI (folinic acid, 5-fluorouracil, oxaliplatin, and irinotecan) plus bevacizumab (independently from molecular subgroups) are the standard regimens with highest activity.^4–7^ Recent data from phase II studies suggest that the combination of triplet chemotherapy with an anti-EGFR agent is feasible and allows achieving impressive response outcomes in molecularly selected patients.^8–10^

Although response parameters including early tumour shrinkage and deepness of response highly influence the chance to achieve R0 resections, the balance of several clinical, molecular and pathologic factors may influence patients’ survival outcomes. Among these latter factors, the histopathologic response to the pre-operative treatment, mainly defined in terms of tumour regression grade (TRG), is crucial.^11–15^ Therefore, the optimal systemic regimen in the setting of liver-limited mCRC should be able to induce not only radiologic, but also histopathologic response. Retrospective studies suggested that the addition of bevacizumab to oxaliplatin-based doublets positively affects the rate of major/complete histopathologic response.^13,16,17^ At the same time, up today no conclusive data about the differential impact of bevacizumab vs anti-EGFRs on TRG were provided, since available series are affected by several bias, including an inappropriate molecular selection of patients treated with anti-EGFRs, and the adoption of heterogeneous chemotherapy backbones.^18,19^

Recently, three different histopathological growth patterns (HGPs) of liver metastases have been described: desmoplastic (i.e., with a capsule of stroma separating tumour and normal cells), pushing (i.e., with limited infiltration of normal hepatic plates by tumour cells), and replacement (i.e., with abundant infiltration of normal hepatic plates by tumour cells and vessel co-option).^20^ From a biologic viewpoint, although metastases with desmoplastic and pushing HGPs rely on angiogenesis for their vascular supply, those with a replacement HGP co-opt pre-existing sinusoidal vessels, suggesting an intrinsically resistance to anti-angiogenic drugs.^21^

Drawing from these considerations, we performed an extensive histopathologic evaluation of CRCLM resected after triplets and either bevacizumab or cetuximab, aiming at evaluating differences in histopathologic parameters of response according to administered targeted agents (bevacizumab vs cetuximab), assessing the independent prognostic impact of histopathologic parameters, and exploring the potential prognostic or predictive role of HGPs.

## Patients and methods

### Study population

From July 2008 to September 2016, 677 mCRC patients received first-line FOLFOXIRI or COI (capecitabine, oxaliplatin and irinotecan) plus bevacizumab or cetuximab in five clinical trials, enrolling patients from 40 Italian Oncology Units. All trials were approved by the local Ethics Committees at all participating centres, and patients provided their written informed consent to receive the treatment and to participate to translational analyses.

TRIBE (NCT00719797; (*N* = 508), 252 in the FOLFOXIRI plus bevacizumab arm),^22^ MOMA (NCT02271464; *N* = 232)^23^ and MACBETH (NCT02295930; *N* = 116)^10^ by Gruppo Oncologico del Nord Ovest (GONO), adopted FOLFOXIRI as chemotherapy backbone; COI-E (EudraCT2008-001062-93; *N* = 31)^9^ and COI-B (NCT02086656; *N* = 46)^24^ by Fondazione IRCCS Istituto Nazionale dei Tumori (INT), used capecitabine, oxaliplatin, and irinotecan (COI). Bevacizumab was the combined targeted agent in TRIBE, MOMA, and COI-B, whereas cetuximab was used in MACBETH and COI-E. For the purpose of the present analysis, among patients treated with cetuximab, only those centrally defined as *RAS* and *BRAF* wild-type were included.

Trials by GONO included untreated mCRC patients, regardless their metastatic sites, with age between 18 and 75 years, ECOG PS of 2 or less (0 for patients between 71 and 75 years old), whose disease was deemed unresectable by experienced multidisciplinary teams. The adoption of guidelines for defining unresectability (i.e., Oncosurge criteria)^25^ was highly recommended and multidisciplinary discussion of resectability was planned at the time of every disease re-assessment. FOLFOXIRI plus bevacizumab or modified FOLFOXIRI plus cetuximab were administered biweekly up to 12 cycles in the TRIBE trial and up to eight cycles in MOMA and MACBETH studies.

Trials by INT included only mCRC patients with borderline resectable liver-limited disease, defined by technical (tumour involvement of >1 hepatic vein or >4 hepatic segments, need for two-stage hepatectomy, portal vein embolisation or intraoperative radiofrequency ablation) and/or biologic reasons (≥4 metastatic nodules, synchronous metastases) predicting high recurrence risk. Four biweekly pre-operative cycles of COI-B or COI-E were planned.

In all studies, disease assessment by contrast-enhanced CT scan of chest and abdomen was performed every 8 weeks until disease progression.

For the purpose of this analysis, we identified patients with liver-limited disease who underwent secondary resection of their metastatic lesions with curative intent and with available tissue samples of resected metastases.

### Histopathologic assessments

All histopathologic assessments were performed by optical microscope and centralised at Fondazione IRCCS Istituto Nazionale dei Tumori, Milan. Tissue samples were independently evaluated by two pathologists (MM, AP) blinded with respect to clinical information, treatment regimen, and outcome. TRG was scored according to the scheme from Mandard et al.,^26^ then modified for liver metastases.^13^ This score identifies five TRGs based on the presence of residual tumour cells and the extent of fibrosis. A cut-off of 3 mm of tumour thickness at the tumour-normal interface (TNI) was used to differentiate minor from major/complete pathologic response.^27^

We distinguished infarct-like necrosis, consisting of large confluent areas of eosinophilic cytoplasmic remnants, located centrally within the lesion and surrounded by fibrosis and foamy macrophages, from tumoural “dirty” necrosis, containing nuclear debris in a patchy distribution.^28^

Lymphocytic intratumoural infiltration and peritumoural inflammatory response were determined using a score ranging from absent (no lymphocytes) to mild (<5 lymphocytes/HPF), moderate (5–10 lymphocytes/HPF), and severe (>10 lymphocytes/HPF).^29,30^

Toxicity-related parameters were evaluated in the non-neoplastic parenchyma and determined based on their presence or absence. Sinusoidal dilatation was graded semiquantitatively as follows: 0, absent; 1, mild (centrolobular involvement limited to one-third of the lobular surface); 2, moderate (centrolobular involvement extending to two-thirds of the lobular surface); 3, severe (complete lobular involvement).

The three common HGPs (desmoplastic, pushing and replacement) were recognised by standard H&E stained tissue sections, according to the key histopathologic characteristics of the growth patterns^20^ (Supplementary Figure 1).

### Statistics

Baseline characteristics and histopathologic parameters of response and toxicity reported in patients treated with triplet plus bevacizumab or triplet plus cetuximab were compared by means of *χ*^2^ test, Fisher exact test or Mann–Whitney test as appropriate.

RFS was calculated from the day of surgical resection to the evidence of disease relapse, or death from any cause. Post-resection OS was calculated from the day of surgical resection until death from any cause. Survival curves were estimated by the Kaplan–Meier method and compared with the log-rank test. The impact of histopathologic response and other prognostic factors on relapse-free survival (RFS) and post-resection overall survival (OS) was firstly assessed in univariate analyses. Significantly prognostic variables (*p* < 0.10) were included in a multivariable Cox proportional hazard model.

We investigated the effects of clinical and molecular characteristics (sex, time between the diagnosis of CRC and the development of metastases, number of liver metastases and involved segments, lobar distribution of liver metastases, longest diameter of liver metastases, primary tumour location, prior primary resection, disease-free interval, CEA levels, *RAS*, and *BRAF* mutational status) and of radiologic response parameters (RECIST response, early response, and deepness of response^31^ on the probability of achieving major histopathologic response in univariate analyses. Odds ratios (OR) and relative 95% confidence intervals (CIs) were calculated. Variables significantly (*p* < 0.10) affecting the probability of undergoing liver surgery were included in a logistic regression model.

The efficacy of bevacizumab vs cetuximab in the different HGPs was assessed in terms of progression-free survival (PFS) that was calculated from the day of study entry (registration or randomisation) to the first observation of disease progression according to RECIST, or death from any cause.

## Results

Liver metastases from 159 patients were analysed. Patients’ baseline characteristics are summarised in Table 1. Most of them had ECOG PS 0 (96%) and presented with synchronous liver metastases (82%). At the time of enrollment, in situ primary tumours were documented in 25% of patients. FOLFOXIRI and COI had been pre-operatively administered as chemotherapy backbones in 92 (58%) and 67 (42%) cases, respectively, and 103 (65%) and 56 (35%) patients had received bevacizumab and cetuximab as targeted agents (Supplementary Figure 2). With the obvious exception of the mutational status, no significant differences between the two treatment subgroups were reported. In the overall population, at a median follow up of 42.1 months, median RFS and median OS were 12.2 and 47.2 months, respectively.

**Table 1:** Patients’ and disease characteristics in the overall population and according to treatment groups

	Overall population *N* (%)	Triplet + bev *N* (%)	Triplet + cetuximab *N* (%)	*p*
	*N* = 159	*N* = 103	*N* = 56	
Baseline characteristics				
Age (range)	60 (23–75)	61 (23–75)	57 (32–70)	—
ECOG PS				
0	152 (96)	98 (95)	54 (96)	
1–2	7 (4)	5 (5)	2 (4)	1.000
Sex				
Male	97 (61)	63 (61)	34 (61)	
Female	62 (39)	40 (39)	22 (39)	1.000
Time to metastases				
Synchronous	131 (82)	86 (83)	45 (80)	
Metachronous	28 (18)	17 (17)	11 (20)	0.666
No. of liver metastases				
≥4	59 (37)	42 (41)	17 (30)	
<4	93 (59)	58 / 56	35 / 63	0.296
NA	7 (4)	3 (3)	4 (7)	
Primary resected				
No	39 (25)	22 (21)	17 (30)	
Yes	120 (75)	81 (79)	39 (70)	0.248
Location of primary tumour				
Right colon	40 (25)	31 (30)	9 (16)	
Left colon	64 (40)	40 (39)	24 (43)	
Extraperitoneal rectum	52 (33)	29 (28)	23 (41)	0.055
NA	3 (2)	3 (3)	0 (0)	
Nodal status of primary tumour				
Node positive	85 (53)	60 (59)	25 (45)	
Node negative	35 (22)	21 (20)	14 (25)	0.356
NA	39 (25)	22 (21)	17 (30)	
Tumour size, diameter				
>5 cm	56 (35)	38 (37)	18 (32)	
≤5 cm	102 (64)	65 (63)	37 (67)	0.603
NA	1 (1)	0 (0)	1 (1)	
Distribution of liver metastases				
Bilobar	96 (60)	63 (61)	33 (60)	
Unilobar	54 (34)	32 (31)	22 (39)	0.482
NA	9 (6)	8 (8)	1 (1)	
No. of involved segments				
≥6	19 (12)	13 (13)	6 (11)	
<6	116 (73)	72 (70)	44 (78)	0.623
NA	24 (15)	18 (17)	6 (11)	
Disease-free interval				
<12 months	137 (86)	90 (87)	47 (84)	
>12 months	22 (14)	13 (13)	9 (16)	0.632
Mutational status				
* RAS/BRAF* wt	91 /(57)	35 (34)	56 (100)	
* RAS* mut	57 (36)	57 (55)	0 (0)	
* BRAF* mut	6 (4)	6 (6)	0 (0)	**<0.001**
NA	5 (3)	5 (5)	0 (0)	

The "p" in bold indicate the p value statistically significant

Table 2 summarises the results in terms of histopathologic evaluations in the overall population and according to the two treatment subgroups. Pathologic complete response was detected in 7 (7%) and 1 (2%) case in the bevacizumab and in the cetuximab group, respectively (*p* = 0.436). In significantly higher percentages of cases in the bevacizumab than in the cetuximab group major histopathologic response (TRG1–2, 32 vs 14%, *p* = 0.013) and infarct-like necrosis (80 vs 64%, *p* = 0.035) were reported. No differences in other parameters of histopathologic response and toxicity were observed (Table 2). In the bevacizumab group, no significant differences between *RAS* and *BRAF* wild-type cases and those bearing any *RAS* or *BRAF* mutation were evident (Supplementary Table 1), although all complete histopathologic responses occurred in *RAS*-mutated tumours.

**Table 2:** Histopathologic parameters in the overall population and according to treatment groups

	Overall population *N* (%)	Triplet + bev *N* (%)	Triplet + cetuximab *N* (%)	*p*
	*N* = 159	*N* = 103	*N* = 56	
Resection margins				
R0	133 (84)	84 (82)	49 (88)	0.378
R1	26 (16)	19 (18)	7 (14)	
pCR				
Yes	8 (5)	7 (7)	1 (2)	0.436
No	151 (95)	96 (93)	55 (98)	
Histopathologic response				
TRG1	8 (5)	7 (7)	1 (2)	**0.013**
TRG2	33 (21)	26 (25)	7 (12)	
TRG3	53 (33)	33 (32)	20 (36)	
TRG4	49 (31)	32 (31)	17 (30)	
TRG5	16 (10)	5 (5)	11 (20)	
Major response (TRG1–2)	41 (26)	33 (32)	8 (14)	**0.015**
Partial response (TRG3)	53 (33)	33 (32)	20 (36)	
No response (TRG4–5)	65 (41)	37 (36)	28 (50)	
Tumour-normal tissue interface				
<3 mm	83 (52)	59 (57)	24 (43)	0.082
>3 mm	76/48	44 (43)	32 (57)	
Necrosis				
Mean	49	49	51	
≥40%	27 (17)	17 (17)	10 (18)	0.823
<40%	132 (83)	86 (83)	46 (92)	
Fibrosis				
Mean	23	24	20	
≥40%	110 (69)	72 (70)	38 (68)	0.791
<40%	49 (31)	31 (30)	18 (32)	
Infarct-like necrosis				
Yes	118 (74)	82 (80)	36 (64)	**0.035**
No	41 (26)	21 (20)	20 (36)	
Lymphocitic infiltration				
Absent	22 (14)	16 (16)	6 (11)	0.726
Mild	119 (75)	76 (74)	43 (78)	
Moderate	17 (11)	11 (10)	6 (11)	
Peritumoural inflammatory response				
Mild	95 (60)	58 (56)	37 (66)	0.373
Moderate	60(38)	41 (40)	19 (34)	
Intense	2 (1)	2 (2)	0 (0)	
NA	2 (1)	2 (2)	0 (0)	
Microvescicular steatosis				
Yes	108 (68)	73 (71)	35 (63)	0.173
No	48 (30)	27 (26)	21 (37)	
NA	3 (2)	3 (3)	0 (0)	
Macrovescicular steatosis				
Yes	72 (46)	44 (43)	28 (50)	0.439
No	85 (53)	57 (55)	28 (50)	
NA	2 (1)	2 (2)	0 (0)	
Sinusoidal dilatation				
0 (absent)	45 (29)	28 (28)	17 (30)	0.932
1 (mild)	56 (35)	35 (34)	21 (38)	
2 (moderate)	41 (25)	28 (27)	13 (23)	
3 (severe)	14 (9)	9 (8)	5 (9)	
NE	3 (2)	3 (3)	0 (0)	
Parenchimal necrosis				
Yes	14 (9)	12 (11)	2 (4)	0.140
No	141 (88)	89 (87)	52 (93)	
NE	4 (3)	2 (2)	2 (3)	
Pericellular fibrosis				
Yes	20 (12)	16 (16)	4 (7)	0.112
No	136 (86)	84 (81)	52 (93)	
NE	3 (2)	3 (3)	0 (0)	

The "p" in bold indicate the p value statistically significant

Among investigated baseline characteristics, radiologic response parameters and treatment subgroups, the administration of bevacizumab instead of cetuximab (OR = 2.83, 95% CI = 1.20–6.65; *p* = 0.015) and of FOLFOXIRI instead of COI (OR = 2.90, 95% CI = 1.30–6.44; *p* = 0.008), and the deepness of radiologic response (OR = 1.31, 95% CI = 1.07–1.60; *p* = 0.009) were significantly associated with the probability of achieving a major histopathologic response (Table 3). In the multivariable model, including the three covariates, only the deepness of response (OR = 1.52, 95% CI = 1.38–1.94; *p* < 0.001) and the administered targeted agent (OR = 6.00, 95% CI = 1.96–18.40; *p* = 0.002) were significantly associated with the probability of achieving a major response.

**Table 3:** Association of baseline characteristics and response parameters with the probability of achieving major histopathologic response

			Univariate analysis			Multivariate analysis		
	*N*	%	OR	95% CI	*p*	OR	95% CI	*p*
Baseline characteristics								
Sex								
Male	97	23.7	1	—	—	—	—	—
Female	62	29.0	1.32	0.64–2.71	0.454	—	—	—
Time to metastases								
Synchronous	131	25.2	1	—	—	—	—	—
Metachronous	28	28.6	1.88	0.48–2.95	0.708	—	—	—
No. of liver metastases								
≥4	59	27.1	1	—	—	—	—	—
<4	74	28.4	1.06	0.50–2.29	0.862	—	—	—
Primary resected								
No	38	36.8	1	—	—	—	—	—
Yes	120	22.5	0.50	0.23–1.09	0.079	—	—	—
Location of primary								
Right colon	40	27.5	1	—	—	—	—	—
Left colon	116	25.0	0.88	0.39–1.98	0.752	—	—	—
Tumour size, diameter								
>5 cm	56	32.1	1	—	—	—	—	—
≤5 cm	102	22.5	0.61	0.30–1.27	0.188	—	—	—
Distribution of liver metastases								
Bilobar	96	19.8	1	—	—	—	—	—
Unilobar	54	29.6	1.71	0.79–3.69	0.171	—	—	—
No. of involved segments								
>6	14	35.7	1	—	—	—	—	—
≤6	121	27.3	0.68	0.21–2.16	0.536	—	—	—
Disease-free interval								
<12 mos	22	22.7	1	—	—	—	—	—
>12 mos	137	26.3	1.21	0.42–3.52	0.729	—	—	—
Mutational status								
All wt	91	19.8	1	—	—	—	—	—
* RAS* mut	54	25.9	1.42	0.64–3.15	0.390	—	—	—
* BRAF* mut	6	33.3	2.03	0.34–11.95	0.600	—	—	—
Targeted agent								
Cetuximab	56	14.3	1	—	—	1	—	—
Bevacizumab	103	32.0	2.83	1.20–6.65	**0.015**	6.00	1.96–18.40	**0.002**
Chemotherapy backbone								
COI	67	14.9	1	—	—	1	—	—
FOLFOXIRI	92	33.7	2.90	1.30–6.44	**0.008**	0.62	0.14–2.64	0.516
Response parameters								
RECIST response								
No	24	16.7	1	—	—	—	—	—
Yes	135	27.4	1.89	0.60–5.89	0.267	—	—	—
Early tumour shrinkage								
No	23	13.0	1	—	—	—	—	—
Yes	126	28.6	2.67	0.75–9.53	0.119	—	—	—
Deepness of response (per 10% increase)	121	—	1.31	1.07–1.60	**0.009**	1.52	1.38–1.94	**<0.001**

The "p" in bold indicate the p value statistically significant

**Fig. 1:**
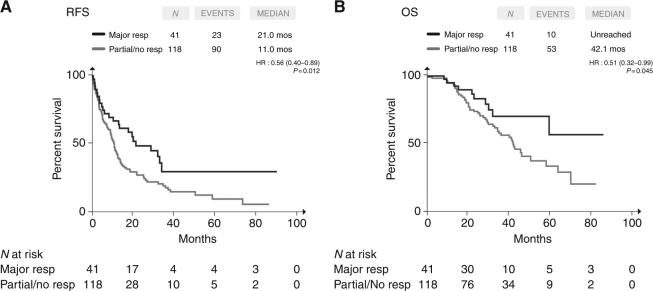
Kaplan–Meier estimates of RFS (**a**) and post-resection OS (**b**) according to the histopathologic response. Major response: TRG1–2; partial/no response: TRG3-4-5

Histopathologic response according to TRG was the only parameter associated with post-resection outcomes (Supplementary Table 2). In fact, when compared with patients reporting partial or no pathologic response (*N* = 118), those achieving major response (*N* = 41) showed significantly longer RFS (median RFS 21.0 vs 11.0 months, HR = 0.56, 95% CI = 0.40–0.89; *p* = 0.012) (Fig. 1a). As shown in Table 4, when adjusting for clinical characteristics associated with RFS at univariate analyses, only the histopathologic response (HR = 0.41, 95% CI = 0.21–0.79; *p* = 0.008), as well as the nodal status of the primary tumour (HR = 0.50, 95% CI = 0.28–0.88; *p* = 0.018), retained its prognostic impact in the multivariable model.

**Table 4:** Association of baseline characteristics, treatment, and response parameters with relapse-free and overall survival

			Univariate analysis	Multivariate analysis		*p*
	*N*	Median	HR for RFS (95% CI)	*p*	HR for RFS (95% CI)	
Baseline characteristics						
>ECOG PS						
0	152	12.2	1		—	
1–2	7	13.4	1.03 (0.39–2.76)	0.953	—	—
Time to metastases						
Synchronous	131	11.0	1		1	
Metachronous	28	19.8	0.59 (0.41–0.96)	**0.034**	0.95 (0.53–1.70)	0.854
No. of liver metastases						
≥4	59	9.3	1		1	
<4	93	19.8	0.62 (0.40–0.91)	**0.018**	0.92 (0.49–1.72)	0.804
NA	7	—	—			
Primary resected						
No	39	9.7	1		1	
Yes	120	13.8	0.61 (0.34–0.91)	**0.020**	0.50 (0.17–1.50)	0.221
Location of primary tumour						
Right colon	40	11.3	1		—	
Left colon	116	12.0	0.81 (0.52–1.23)	0.301	—	—
NA	3	—	—		—	
Nodal status of primary tumour						
Node positive	85	12.6	1		1	
Node negative	35	37.1	0.53 (0.36–0.85)	**0.009**	0.50 (0.28–0.88)	**0.018**
NA	39	—	—		—	
Tumour size, diameter						
>5 cm	56	11.0	1		—	
≤5 cm	102	12.7	1.00 (0.68–1.48)	0.993	—	—
NA	1	—	—		—	
Distribution of liver metastases						
Bilobar	96	10.4	1		1	
Unilobar	54	16.7	0.68 (0.47–1.01)	**0.058**	0.95 (0.52–1.72)	0.864
NA	9	—	—			
No. of involved segments						
>6	19	9.2	1		—	
≤6	116	13.8	0.71 (0.32–1.37)	0.273	—	—
NA	24	—	—		—	
Disease-free interval						
<12 months	137	11.3	1		—	
>12 months	22	13.3	0.88 (0.54–1.46)	0.220	—	—
Mutational status						
* RAS/BRAF* wt	91	12.6	1		—	
* RAS* mut	57	12.7	0.93 (0.63–1.38)	0.729	—	—
* BRAF* mut	6	2.4	1.99 (0.66–10.57)	0.170	—	—
NA	5	—	—			
CEA						
<200 ng/ml	112	11.0	1		—	
>200 ng/ml	21	13.8	0.91 (0.50–1.63)	0.740	—	—
NA	26	—	—			
Treatment						
Targeted agent						
Cetuximab	56	10.4	1		—	
Bevacizumab	103	12.7	0.87 (0.59–1.27)	0.463	—	—
Targeted agent (wt only)						
Cetuximab	56	10.4	1		—	
Bevacizumab	35	21.5	0.67 (0.41–1.14)	0.151	—	—
Chemotherapy backbone						
FOLFOXIRI	92	16.7	1		1	
COI	67	17.7	0.64 (0.45–0.94)	**0.022**	0.74 (0.44–1.26)	0.277
Response parameters						
RECIST response						
>No	24	5.9	1		1	
Yes	134	13.4	0.53 (0.29–0.97)	**0.040**	0.67 (0.36–1.28)	0.231
NA	1	—	—			
Early response						
No	23	4.9	1		—	
Yes	125	13.0	0.52 (0.23–0.72)	**0.005**	—	—
NA	11	—	—			
Deepness of response						
Per 10% increase	121	—	0.96 (0.87–1.05)	0.350	—	—
Tumour regression grade						
Partial/no histopathologic response (TRG3–4–5)	118	11.0	1		1	
Major histopathologic response (TRG1–2)	41	21.0	0.56 (0.40–0.89)	**0.012**	0.41 (0.21–0.79)	**0.008**
NA	1	—				
Resection margins						
R0	133	12.7	1		—	
R1	26	10.4	1.02 (0.63–1.67)	**0.931**	—	—
Baseline characteristics						
ECOG PS						
0	152	46.0	1		1	
1–2	7	23.3	2.86 (1.19–31.75)	**0.032**	6.25 (0.67–58.09)	0.109
Time to metastases						
Synchronous	131	41.6	1		1	
Metachronous	28	Undef	0.53 (0.33–1.04)	**0.068**	0.97 (0.27–3.43)	0.958
No. of liver metastases						
≥4	59	34.8	1		1	
<4	93	51.0	0.50 (0.29–0.83)	**0.008**	1.20 (0.32–4.41)	0.789
NA	7	—	—		—	
Primary resected						
No	39	33.4	1		1	
Yes	120	44.6	0.59 (0.28–0.99)	**0.051**	0.84 (0.09–7.84)	0.879
Location of primary tumour						
Right colon	40	42.7	1		—	
Left colon	116	43.2	0.96 (0.55–1.70)	0.896	—	
NA	3	—	—		—	
Nodal status of primary tumour						
Node positive	85	42.4	1		1	
Node negative	35	58.6	0.54 (0.31–1.07)	**0.080**	0.45 (0.14–1.42)	0.175
NA	39	—	—		—	
Tumour size, diameter						
>5 cm	56	36.5	1		—	
≤5 cm	102	58.6	0.69 (0.40–1.14)	0.143	—	
NA	1	—	—		—	
Distribution of liver metastases						
Bilobar	96	36.5	1		**1**	
Unilobar	54	58.6	0.52 (0.32–0.91)	**0.021**	0.69 (0.24–1.98)	0.496
NA	9	—	—			
No. of involved segments						
>6	19	33.4	1		**1**	
≤6	116	46.6	0.43 (0.11–0.79)	**0.016**	2.06 (0.36–11.89)	0.422
NA	24	—	—		—	
Disease-free interval						
<12 months	137	42.4	1		—	
>12 months	22	43.2	0.82 (0.43–1.61)	0.589	—	—
Mutational status						
* RAS/BRAF* wt	91	46.0	1		1	
* RAS* mut	57	42.7	1.16 (0.67–2.03)	0.580	1.77 (0.80–3.94)	0.873
* BRAF* mut	6	18.7	6.71 (2.36–180.20)	**<0.001**	3.80 (0.30–210.50)	0.632
NA	5	—	—			
CEA						
<200 ng/ml	112	34.9	1		1	
>200 ng/ml	21	58.6	0.57 (0.22–1.10)	**0.089**	0.48 (0.15–1.54)	0.220
NA	26	—	—		—	
Treatment						
Targeted agent						
Cetuximab	56	46.6	1		—	
Bevacizumab	103	42.4	1.21 (0.75–2.00)	0.445	—	—
Targeted agent (wt only)						
Cetuximab	56	46.6	1		—	
Bevacizumab	35	34.8	1.08 (0.50–2.35)	0.839	—	—
Chemotherapy backbone						
FOLFOXIRI	92	36.5	1		1	
COI	67	64.3	0.52 (0.33–0.90)	**0.019**	0.94 (0.27–3.31)	0.928
RECIST response						
No	24	29.5	1		1	
Yes	134	46.0	0.58 (0.23–1.13)	**0.098**	0.51 (0.17–1.55)	0.239
NA	1	—	—			
Early response						
No	23	21.3	1		—	
Yes	125	46.0	0.45 (0.16–0.74)	**0.006**	—	—
NA	11	—	—			
Deepness of response						
Per 10% increase	121	—	0.95 (0.84–1.06)	0.343	—	—
Histopathologic response						
Partial/no response (TRG3–4–5)	118	42.1	1		1	
Major response (TRG1–2)	41	Undef	0.51 (0.32–0.99)	**0.045**	0.26 (0.07–0.89)	**0.033**
NA	1	—				
Resection margins						
R0	133	42.7	1			
R1	26	70.1	0.67 (0.37–1.34)	0.284		

The "p" in bold indicate the p value statistically significant

Consistently, major response was associated with longer OS (median OS: unreached vs 42.1 months, HR = 0.51, 95% CI = 0.32–0.99; *p* = 0.045) (Fig. 1b). In the multivariable model (Table 4), the histopathologic response was the only variable independently associated with OS (HR = 0.26, 95% CI = 0.07–0.89, *p* = 0.033).

**Fig. 2:**
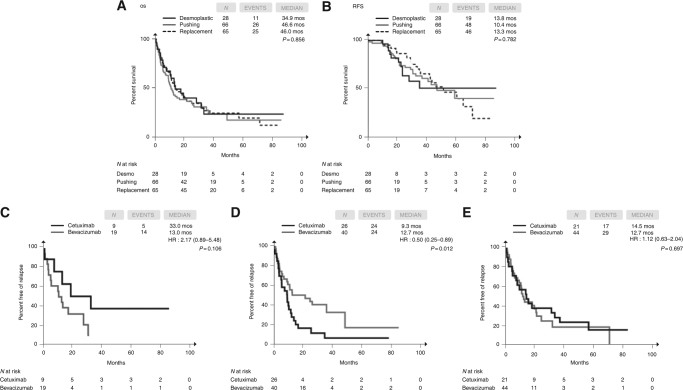
Kaplan–Meier estimates of post-resection OS (**a**) and RFS (**b**) according to HGPs in the overall population and of RFS in the desmoplastic (**c**), pushing (**d**) and replacement (**e**) HGPs according to the administered targeted agent

Desmoplastic, pushing, and replacement HGPs were found in 28 (18%), 66 (41%), and 65 (41%) specimens, respectively. In the overall population, no impact of HPGs on survival parameters was observed (OS log-rank *p* = 0.856; RFS log-rank *p* = 0.783) (Fig. 2a, b), but a higher percentage of cases with desmoplastic HGP showed a major histopathologic response (57 vs 17% in pushing and 22% in replacement HGP, *p* < 0.001). Although among patients whose metastases presented a desmoplastic HGPa non-significant advantage for cetuximab was reported (HR = 2.17, 95% CI = 0.89–5.48, *p* = 0.106; Fig. 2c), significantly longer RFS was achieved with bevacizumab than with cetuximab in the pushing subgroup (HR = 0.50, 95% CI = 0.25–0.84, *p* = 0.012; Fig. 2d). In the replacement subgroup, no differences between the two agents were reported (RFS: HR = 1.12, 95% CI = 0.63–2.04, *p* = 0.697) (Fig. 2e). Consistent results were achieved when the analyses were restricted to *RAS* and *BRAF* wild-type patients (Supplementary Figure 3a–c). The association of HGPs with histopathologic response according to the administered targeted agent is described in the Supplementary Figure 4.

## Discussion

The landscape of CRCLM is extremely heterogeneous and multiple clinical, molecular, and pathological factors weight on patients’ outcomes.^32^ Because of the wide spectrum of potential clinical scenarios, both the design of clinical studies and the applicability of trials’ results in the daily practice are extremely difficult. In fact, in spite of the limited amount of prospective evidence in this field, the management of patients with CRCLM has notably changed in the last decade, with a clinically meaningful absolute survival gain.^33^ Not only the improvements in innovative surgical and other locoregional techniques, but also the availability of several conversion or neoadjuvant systemic regimens contributed to these advances, that are expected to further improve over time.^22,34–37^ Increasing evidence is collected about the possibility to significantly affect the natural history of the disease through a substantial pharmacological and surgical debulking of the tumour burden, made possible by the multidisciplinary management of affected patients.

To this regard, the pathologists’ role in the multidisciplinary team is increasingly important, although further effort is needed to clarify how to exploit histopathologic parameters to drive treatment decisions. Here we confirm the impact of TRG on the post-operative outcome, while no association of necrosis, fibrosis and infarct-like necrosis with survival is reported.^11,13,15–17^

It is arguable that TRG mirrors the ability of pre-operative regimens to control the micrometastatic disease, thus reducing the probability to experience disease relapse.

Our data strengthen this concept, since the association between TRG and survival parameters is retained in the multivariable model, aiming at catching the heterogeneity of potential clinical presentations and previous responses to systemic treatments. As all patients included in the present analysis received a triplet plus a biologic as pre-operative treatment, it seems that the use of highly active regimens as conversion or neoadjuvant treatments may counterbalance the poor prognostic impact of negative baseline characteristics when achieving a radical resection of metastatic lesions. In other words, clinical and molecular factors weighing on post-resection outcomes are no longer relevant when using these upfront treatments. On the other side, achieving a relevant histopathologic response is a major determinant of clinical outcome and, notably, is significantly associated with deeper radiologic response. The possibility to adopt different post-operative strategies based on histopathologic response results is worth of investigation in properly designed prospective trials.

Here we also show that the use of triplets plus bevacizumab had more histopathologic responses than triplets plus cetuximab. Up today, whereas different retrospective analyses with several potential biases consistently suggested that the addition of bevacizumab to chemotherapy alone increases the rate of major histopathologic responses, conflicting results were provided with regard to the comparison of chemotherapy plus either bevacizumab or an anti-EGFR.^18^^,^^19^ Notably, all these studies were invariably biased by their retrospective nature, the heterogeneity of chemotherapy backbones and the inappropriate molecular selection of patients treated with the anti-EGFRs.

Our effort suffers of some limitations. Firstly, trials included in our pooled analysis did not randomise between bevacizumab and cetuximab. However, inclusion criteria of these studies were perfectly superimposable, with the exception of the molecular selection for studies evaluating cetuximab-containing regimens, and the studies were conducted in the same timeframe. As a consequence, characteristics of enrolled patients were highly balanced in the two groups. Secondly, only patients with initially unresectable or borderline resectable but at high risk of recurrence are included, thus preventing from applying present results to easily resectable patients at low risk of recurrence. However, these patients are not candidate to receive biologic agents, and in particular anti-EGFRs since a potential detrimental effect with the addition of cetuximab to perioperative oxaliplatin-based doublets was evidenced.^33^ Thirdly, although the homogeneity of chemotherapy backbones definitely represents a strong point of this analysis, 31 patients received capecitabine as part of the chemotherapy regimen, in combination with cetuximab. The association of fluoropyrimidines other than infusional 5-fluoruracil with anti-EGFR monoclonal antibodies is not recommended by current guidelines. Nevertheless, in the multivariable model the impact of the targeted agent on the probability of achieving a major histopathologic response is independent of the associated chemotherapy regimen. Finally, we were not able to provide formal demonstration that regimens able to determine better histopathologic responses favorably affect survival, thus failing to prove the surrogacy of TRG for OS, as other previous series in this field did.

The evaluation of CRCLM’ HGPs has recently gained attention from the oncology perspective due to its potential prognostic and even predictive meaning, as well as its easy assessment in H&E stained slides.^20,21^ The available retrospective literature suggests that replacement HGP may be associated with poorer prognosis, worse histopathologic response to neoadjuvant treatments and lack of survival benefit from the addition of bevacizumab to chemotherapy alone. To the best of our knowledge, this is the first attempt to potentially catch a differential benefit from bevacizumab vs cetuximab according to HGPs. Even if our series cannot provide definitive conclusions, interesting results about the better efficacy of bevacizumab and cetuximab in the pushing and in the desmoplastic patterns, respectively, were found and should be validated through properly designed randomised trials. The lack of prognostic impact of HGPs in the present series, differently from literature data, may be explained by the fact that the adopted highly active pre-operative regimens may have weakened the weight of poor prognostic factors including replacement HPG. A similar effect was previously shown by our group about the lack of negative impact of *BRAF* mutation in mCRC patients with LLD, resected after FOLFOXIRI plus bevacizumab.^7^ Moreover, the use of systemic treatments may somehow change the percentage of a specific HGPs component in favor of another thus representing a potential confounding effect in our and previous studies, which mostly included patients treated in the pre-operative setting. Finally, assessing HGPs post-operatively clearly hampers its potential application to the choice of the pre-operative strategy. Therefore, to deepen and hopefully translate to clinical practice the predictive power of HGPs, additional valuable information should be prospectively obtained through liver biopsies performed before starting the conversion/neoadjuvant treatment. To this purpose, the possibility to classify accurately HGPs by means of pre-treatment imaging parameters should be investigated.

## Electronic supplementary material

Supplementary figure 1

SUPPLEMENTARY table 1

SUPPLEMENTARY table 2

SUPPLEMENTARY FIGURE 2

SUPPLEMENTARY FIGURE 3

SUPPLEMENTARY FIGURE 4

Color form
