# Supplementary figures and images for: Differential histopathologic parameters in colorectal cancer liver metastases resected after triplets plus bevacizumab or cetuximab: a pooled analysis of five prospective trials
Source: Br J Cancer. 2018 Mar 13;118(7):955–65. doi: 10.1038/s41416-018-0015-z (PMC5931102; doi:10.1038/s41416-018-0015-z)

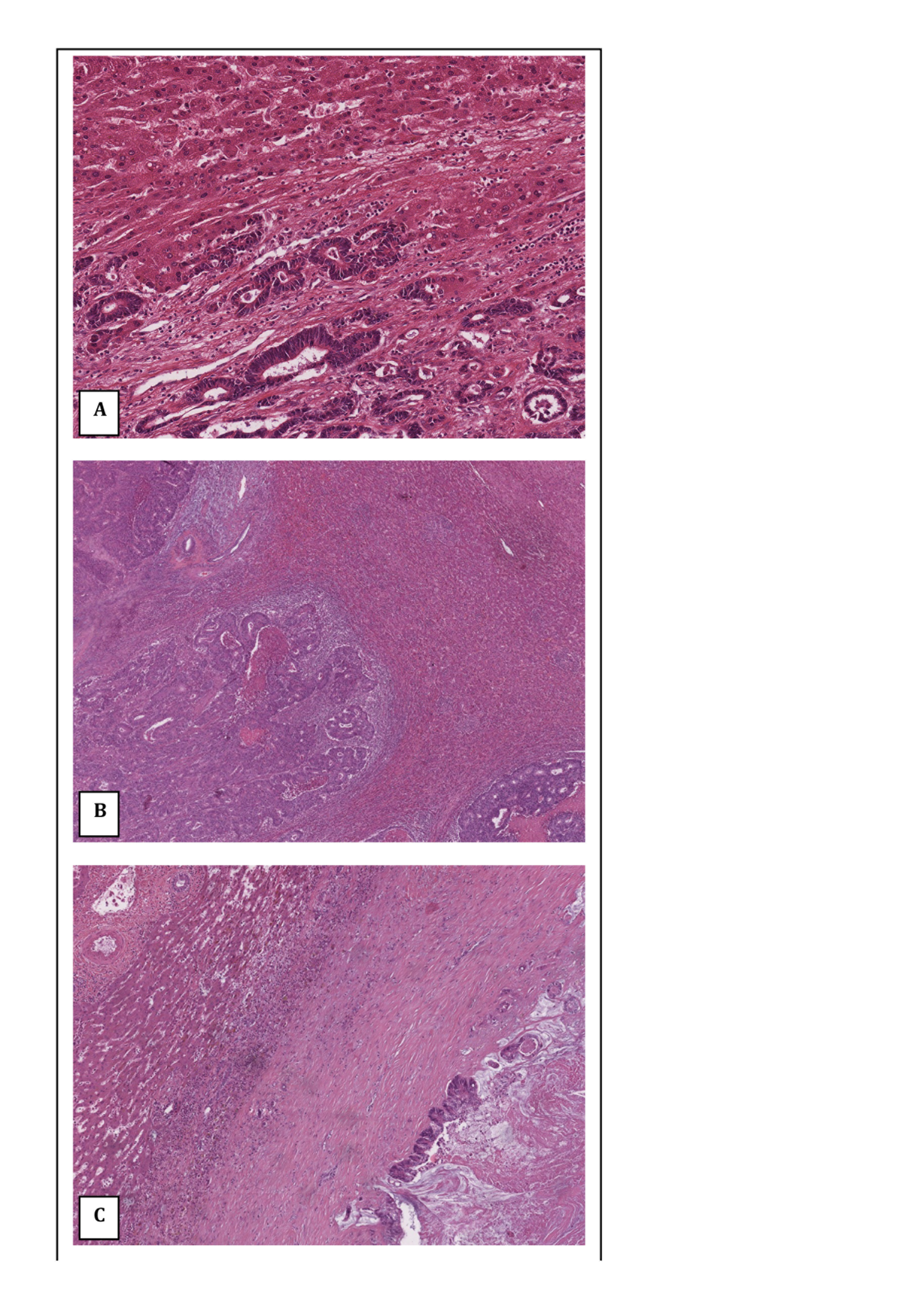

Supplement: Supplementary file 1 — Supplementary figure 1 [file 41416_2018_15_MOESM1_ESM.docx]

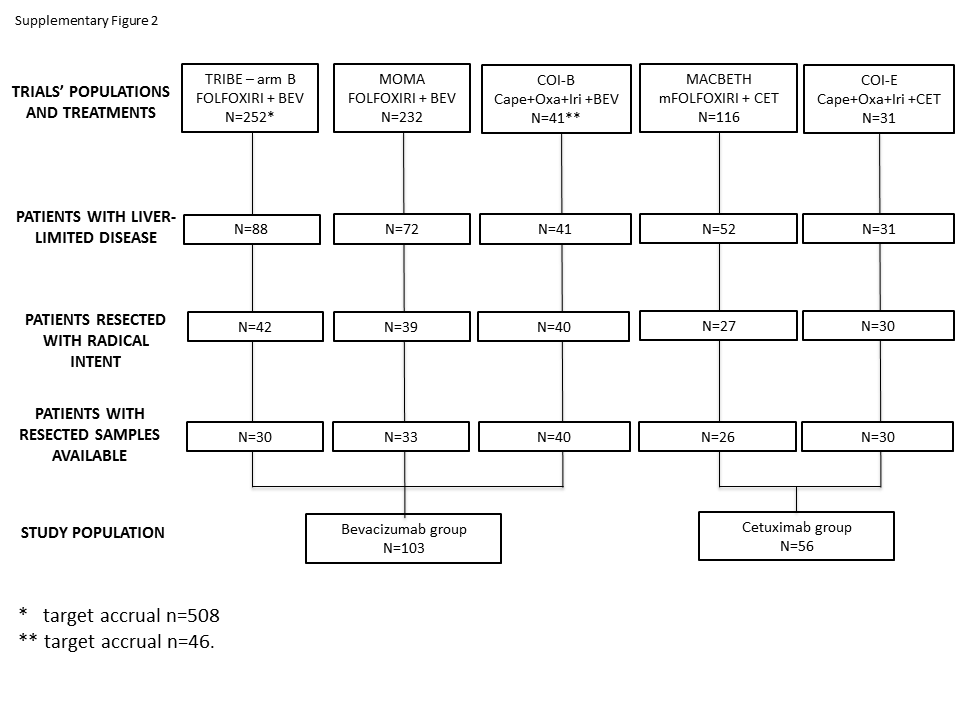

Supplement: Supplementary file 4 — SUPPLEMENTARY FIGURE 2 [file 41416_2018_15_MOESM4_ESM.tif]

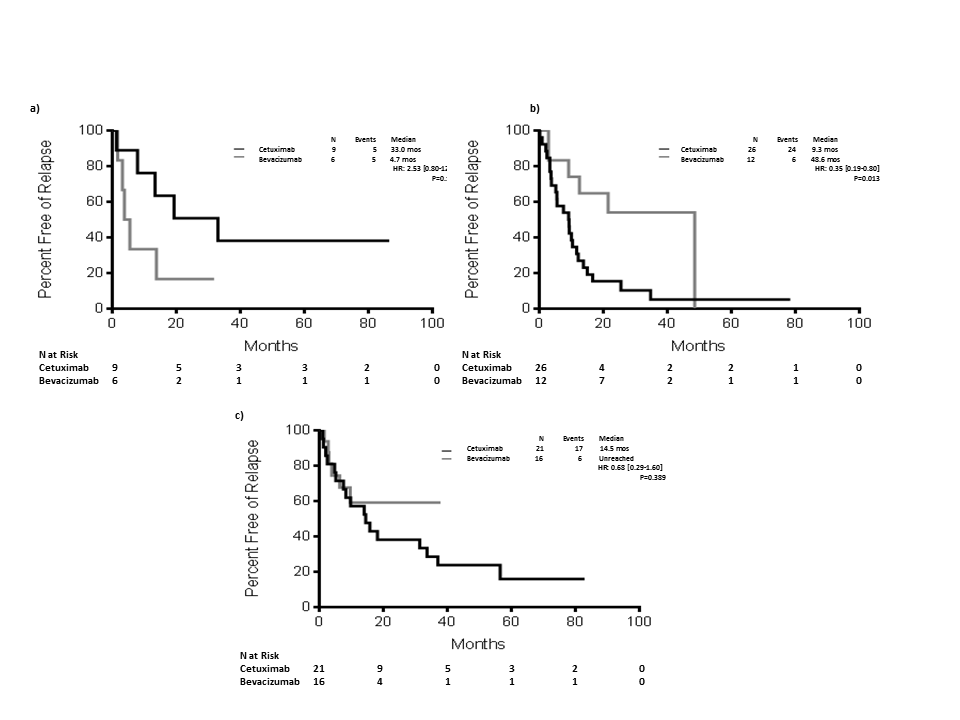

Supplement: Supplementary file 5 — SUPPLEMENTARY FIGURE 3 [file 41416_2018_15_MOESM5_ESM.tif]

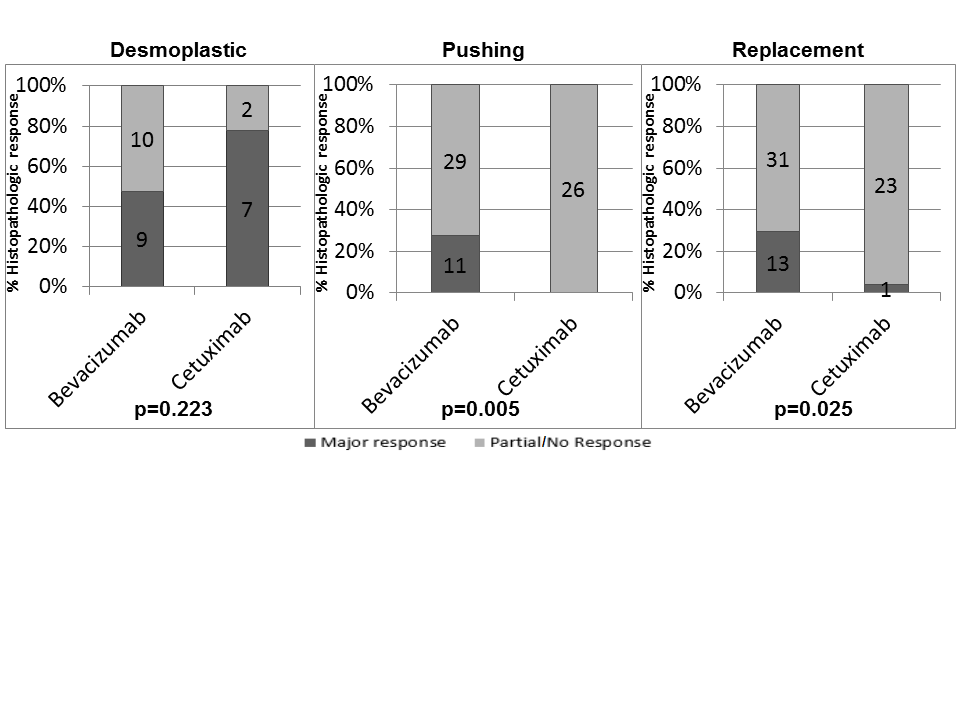

Supplement: Supplementary file 6 — SUPPLEMENTARY FIGURE 4 [file 41416_2018_15_MOESM6_ESM.tif]
