# Supplementary material for: Differential histopathologic parameters in colorectal cancer liver metastases resected after triplets plus bevacizumab or cetuximab: a pooled analysis of five prospective trials
Source: Br J Cancer. 2018 Mar 13;118(7):955–65. doi: 10.1038/s41416-018-0015-z (PMC5931102; doi:10.1038/s41416-018-0015-z)
Supplement: Supplementary file 2 — SUPPLEMENTARY table 1 [file 41416_2018_15_MOESM2_ESM.docx]

|  |  | **Triplet plus bevacizumab**  ***RAS* or *BRAF* mutant**  **(N / %)** | **Triplet plus bevacizumab**  ***RAS* and *BRAF* wt**  **(N / %)** | **P** |
| --- | --- | --- | --- | --- |
|  |  | **N=63** | **N=35** |  |
| *pCR* | yes | 6/10 | 0/0 | 0.086 |
|  | no | 57/90 | 35/100 |  |
| *Histopathologic response* | TRG1 | 6/10 | 0 | 0.174 |
|  | TRG2 | 13/21 | 10/28 |  |
|  | TRG3 | 23/36 | 9/26 |  |
|  | TRG4 | 19/30 | 13/37 |  |
|  | TRG5 | 2/3 | 3/9 |  |
|  | Major response (TRG1-2) | 19/30 | 10/28 | 0.862 |
|  | Partial response (TRG3) | 23/37 | 9/26 |  |
|  | No response (TRG4-5) | 21/33 | 16/46 |  |
| *Tumor-normal tissue interface* | < 3mm | 37/59 | 18/51 | 0.484 |
|  | >3mm | 26/41 | 17/49 |  |
| *Necrosis* | Mean | 19 | 22 |  |
|  | ≥40% | 10/16 | 7/20 | 0.603 |
|  | <40% | 53/84 | 28/80 |  |
| *Fibrosis* | Mean | 48 | 50 |  |
|  | ≥40% | 43/68 | 26/74 | 0.532 |
|  | <40% | 20/32 | 9/26 |  |
| *Infarct-like necrosis* | Yes | 46/73 | 31/89 | 0.072 |
|  | No | 17/27 | 4/11 |  |
| *Lymphocitic infiltration* | Absent | 9/14 | 6/17 | 0.928 |
|  | Mild | 47/75 | 25/72 |  |
|  | Moderate | 7/11 | 4/11 |  |
| *Peritumoral inflammatory response* | Mild | 45/71 | 25/76 | 0.810 |
|  | Moderate | 17/27 | 8/24 |  |
|  | Intense | 1/2 | 0/0 |  |
|  | NA | 0 | 2 |  |
| *Microvescicular steatosis* | Yes | 45/71 | 23/66 | 1.000 |
|  | No | 18/29 | 9/26 |  |
|  | NA | 0 | 2/8 |  |
| *Macrovescicular steatosis* | Yes | 27/43 | 16/48 | 0.597 |
|  | No | 36/57 | 17/52 |  |
|  | NA | 0 | 2 |  |
| *Sinusoidal dilatation* | 0 (absent) | 20/32 | 7/22 | 0.578 |
|  | 1 (mild) | 23/36 | 11/34 |  |
|  | 2 (moderate) | 16/26 | 10/31 |  |
|  | 3 (severe) | 4/6 | 4/13 |  |
|  | NV | 0 | 3 |  |
| *Parenchimal necrosis* | Yes | 7/11 | 4/12 | 1.000 |
|  | No | 56/89 | 29/88 |  |
|  | NV | 0 | 2 |  |
| *Pericellular fibrosis* | Yes | 9/14 | 4/12 | 1.000 |
|  | No | 54/86 | 29/88 |  |
|  | NV | 0 | 2 |  |

**Supplementary Table 1.** Results of assessments on histopathologic parameters in bevacizumab-treated patients according to mutational status (*RAS* and *BRAF* wild-type *versus* *RAS* or *BRAF* mutated).
