# Supplementary material for: Differential histopathologic parameters in colorectal cancer liver metastases resected after triplets plus bevacizumab or cetuximab: a pooled analysis of five prospective trials
Source: Br J Cancer. 2018 Mar 13;118(7):955–65. doi: 10.1038/s41416-018-0015-z (PMC5931102; doi:10.1038/s41416-018-0015-z)
Supplement: Supplementary file 3 — SUPPLEMENTARY table 2 [file 41416_2018_15_MOESM3_ESM.docx]

|  | |  | **Relapse Free Survival** | | | **Overall Survival** | | |
| --- | --- | --- | --- | --- | --- | --- | --- | --- |
|  |  | **N** | **Median** | **HR for RFS [95%CI]** | **p** | **Median** | **HR for OS [95%CI]** | **p** |
| ***Histopathologic parameters of response*** | |  |  |  |  |  |  |  |
| *Histopathologic response* | |  |  |  |  |  |  |  |
| Partial/No response (TRG 3-4-5) | | 118 | 11.0 | 1 |  | 42.1 | 1 |  |
| Major response (TRG 1-2) | | 41 | 21.0 | 0.56 [0.40-0.89] | **0.012** | Undef | 0.51 [0.32-0.99] | **0.045** |
| *Pathologic complete response* | |  |  |  |  |  |  |  |
| No | | 151 | 11.3 | 1 |  | 43.2 | 1 |  |
| Yes | | 8 | 21.0 | 0.48 [0.28-1.19] | 0.141 | Undefined | 0.86 [0.29-2.58] | 0.802 |
| *Necrosis* | |  |  |  |  |  |  |  |
| <40% | | 132 | 11.7 | 1 |  | 43.2 | 1 |  |
| ≥40% | | 27 | 12.6 | 1.12 [0.67-1.84] | 0.180 | Undefined | 0.84 [0.43-1.64] | 0.615 |
| *Fibrosis* | |  |  |  |  |  |  |  |
| <40% | | 49 | 9.8 | 1 |  | 42.1 | 1 |  |
| ≥40% | | 110 | 13.3 | 0.75 [0.49-1.12] | 0.156 | 46.6 | 0.70 [0.39-1.17] | 0.167 |
| *Infarct-like necrosis* | |  |  |  |  |  |  |  |
| No | | 41 | 11.3 | 1 |  | 70.6 | 1 |  |
| Yes | | 118 | 12.7 | 0.99 [0.65-1.51] | 0.968 | 42.7 | 1.02 [0.57-1.80] | 0.954 |
| *Tumor-normal tissue interface* | |  |  |  |  |  |  |  |
| >3mm | | 76 | 11.3 | 1 |  | 42.1 | 1 |  |
| <3mm | | 83 | 13.0 | 0.82 [0.57-1.19] | 0.291 | 60.0 | 0.61 [0.37-1.07] | 0.101 |

**Supplementary Table 2.** Results of univariate analyses investigating the influence of candidate histopathologic parameters on relapse-free survival and overall survival.
