# Supplementary material for: Differential histopathologic parameters in colorectal cancer liver metastases resected after triplets plus bevacizumab or cetuximab: a pooled analysis of five prospective trials
Source: Br J Cancer. 2018 Mar 13;118(7):955–65. doi: 10.1038/s41416-018-0015-z (PMC5931102; doi:10.1038/s41416-018-0015-z)
Supplement: Supplementary file 7 — Color form [file 41416_2018_15_MOESM7_ESM.docx]

**Supplementary online material**

**Supplementary Table 1.** Histopathologic parameters in metastases resected after triplets plus bevacizumab according to mutational status (*RAS* and *BRAF* wild-type versus *RAS* or *BRAF* mutated).

**Supplementary Table 2.** Association of candidate histopathologic parameters with relapse-free and overall survival.

**Supplementary Figure 1.** A. Hematoxylin Eosin at x 100 magnification, histophatological growth pattern Replacement: hepatocytes replaced by cancer cells, incorporating pre-existing vessels from surrounding tissue (vessel co-option).

B. Hematoxilin Eosin at x 40 magnification, histophatological growth pattern Pushing: hepatocytes laminae pushed aside by neoplastic cells.

C. Hematoxilin Eosin at x 40 magnification, histophatological growth pattern Desmoplastic: desmoplastic rim between liver and metastatic tissue.

**Supplementary Figure 2.** Flow diagram showing the composition of study population

**Supplementary Figure 3.** Kaplan Meier estimates of RFS in the *RAS* and *BRAF* wild-type subgroup in the desmoplastic (a), pushing (b) and replacement (c) HGPs according to the administered targeted agent.

**Supplementary Figure 4.** Histopathologic response according to HGPs in the overall population. Major response: TRG1-2; Partial response: TRG 3; No response: TRG 4-5.
